# Slope Identification and Decision Making: A Comparison of Linear and Ratio Graphs

**DOI:** 10.1177/01454455221130002

**Published:** 2022-11-13

**Authors:** Richard M. Kubina, Seth A. King, Madeline Halkowski, Shawn Quigley, Tracy Kettering

**Affiliations:** 1 The Pennsylvania State University, University Park, USA; 2 University of Iowa, USA; 3 Melmark, Berwyn, PA, USA; 4 Bancroft, Cherry Hill, NJ, USA

**Keywords:** trend lines, ratio graphs, linear graphs, decision making, slope identification

## Abstract

Applied behavior analysts have traditionally relied on visual analysis of graphic data displays to determine the extent of functional relations between variables and guide treatment implementation. The present study assessed the influence of graph type on behavior analysts’ (*n* = 51) ratings of trend magnitude, treatment decisions based on changes in trend, and their confidence in decision making. Participants examined simulated data presented on linear graphs featuring equal-interval scales as well as graphs with ratio scales (i.e., multiply/divide or logarithmic vertical axis) and numeric indicators of celeration. Standard rules for interpreting trends using each display accompanied the assessment items. Results suggested participants maintained significantly higher levels of agreement on evaluations of trend magnitude and treatment decisions and reported higher levels of confidence in making decisions when using ratio graphs. Furthermore, decision making occurred most efficiently with ratio charts and a celeration value. The findings have implications for research and practice.

Single-case experimental design (SCED) encompasses a range of within-participant experimental methodologies (e.g., ABAB design, multiple-baseline design) that involve repeated assessment over time and the replication of intervention effects across conditions, individuals, or groups (Kazdin, 2021a). Strongly associated with behavior analysis, research involving SCED also appears in disciplines where the absence of sufficient sample sizes or the emphasis on performance at the individual level (e.g., special education) renders group experimental designs impossible or impractical (Hurtado-Parrado & López-López, 2015). The detection of experimental effects in SCED occurs through visual analysis of data displayed on a linear graph in which the vertical axis (i.e., *y*-axis) depicts changes in an outcome and the horizontal axis (i.e., *x*-axis) depicts a unit of time (Cooper et al., 2020; Kazdin, 2021a).

Visual analysis requires an examination of level (e.g., relation of data to the vertical axis), trend, stability (e.g., consistency of data over time), overlap (i.e., extent to which values maintain across separate conditions), and immediacy of change (i.e., time until apparent effect of intervention; Cooper et al., 2020; Horner & Spaulding, 2010; Kazdin, 2021b). Assessment of intervention effects eschews formal statistical tests, instead relying upon visual comparison of data in one condition (e.g., non-treatment) to an adjacent condition (e.g., treatment) as a means of determining a functional relation (i.e., causal link between changes in the dependent variable and introduction of an intervention; Barton et al., 2018; Johnston et al., 2020). Visual analysis appears on the list of foundational skills applied behavior analysts must master before obtaining certification (Behavior Analyst Certification Board, 2017) and represents a critical competency among researchers and practitioners who use analogs of SCED.

Despite the traditional emphasis on visual analysis in behavior analysis and related fields, researchers have increasingly questioned its technical adequacy and utility as a means of making treatment decisions in practice (e.g., Ninci et al., 2015; Riley-Tillman et al., 2020). Researchers also suggest the limitations of visual analysis may relate to graphing conventions (Kinney et al., 2022). Graphs used in practice typically have horizontal axes that show time linearly (i.e., equal amounts of space indicate equivalent amounts of change in time). However, researchers and practitioners in behavior analysis and related fields typically rely on *linear graphs* (i.e., equal interval graphs) with a vertical axis scaled linearly. In other words, equal distance or space on the vertical axis shows equal *amounts* of change (Schmid, 1986, 1992).

Though relatively less prominent, practitioners in fields complementary to behavior analysis (e.g., precision teaching) have traditionally employed variations of *ratio graphs* (i.e., semilogarithmic graphs), in which equal distance or space between two values on the vertical axis represents an equal ratio of change. Comparisons between the two graphing formats has produced mixed results. Some of the results favor linear graphs while others demonstrate an advantage for ratio graphs (Bailey, 1984; Fuchs & Fuchs, 1986; Kinney et al., 2022; Lefebre et al., 2008; Mawhinney & Austin, 1999). However, researchers have yet to examine the effect ratio graphs, quantification of slope (i.e., trend or celeration line and celeration value), and associated interpretation rules have on decision making. The current article describes the limitations of visual analysis as applied to linear graphs in research and practice. We then the relative advantages of linear and ratio graphs. Finally, we describe the results of a survey study examining the influence of graphing format and associated decision-making rules on practitioners’ interpretation of graphic displays.

## Issues with Traditional Visual Analysis

Although long considered the gold standard for analyzing SCED data (e.g., Manolov et al., 2016), recent developments suggest the dominance of visual analysis in fields associated with SCED (e.g., special education, applied behavior analysis; Hurtado-Parrado & López-López, 2015) has begun to waver. The What Works Clearinghouse (WWC, 2020), the leading research evaluation initiative of the US Department of Education, recently signaled a greater acceptance of SCED by removing the pilot designation from their design standards. In a departure from previous iterations of their SCED evaluation standards, however, the WWC explicitly limited the role of visual analysis in favor of statistically derived measures of effect comparable to those used in traditional group designs. Behavior analysts have a long tradition of rejecting statistical approaches to SCED (e.g., Baer, 1977; Graf, 1982; Johnston et al., 2020; Perone, 1999; Sidman, 1960); nonetheless, supplements to visual analysis have become far more acceptable within the field in recent years (e.g., Hantula, 2016; Killeen, 2019; Kyonka et al., 2019).

Openness to substitutes for visual analysis likely stems from the body of evidence concerning its unreliability and adverse sensitivity (Ninci et al., 2015). Factors such as the expertise of the research team (Hojem & Ottenbacher, 1988), data characteristics (e.g., variability; Van Norman & Christ, 2016), and study context (e.g., social significance of the dependent variable; Ottenbacher, 1990) can lead to disparate interpretations of the same graph. Research increasingly suggests elements of the graphic display, such as the scale depicted on the vertical axis or the relative length of axes, may also distort visual analysis (Dart & Radley, 2017; Radley et al., 2018). Low agreement poses serious questions for the use of visual analysis as a means of supporting the effectiveness of interventions. The absence of transparent or consistent guidelines for the procedure in the research literature represents an additional explanation for the lack of consensus among analysts may occur due to (Barton et al., 2019; King et al., 2020).

Many authors have advocated for more explicit descriptions of techniques associated with visual analysis to appear in published research (i.e., systematic visual analysis; Ninci et al., 2015; Nelson et al., 2017). Lane and Gast (2014) describe approaches for operationalizing aspects of visual design, such as the stability envelope, which determines the presence of variability within a data path by identifying the number of observations within ±25% of the median. Analysts further suggest supplementing qualitative assessments of trend with the split-middle method, which involves bisecting a data path, identifying the midpoint of each segment, and drawing a line through the median vertical axis value for each segment at each midpoint. Calculating the relative level change, which involves subtracting the median vertical axis value from the first data path segment from the median of the second data path segment, represents an additional quantitative approach to evaluating trend.

Although transparent approaches to visual analysis may hold promise in terms of increasing agreement among researchers and their audiences, such procedures may have limited relevance for practitioners. For example, the emphasis on identifying a functional relation represents a major limitation of visual analysis (Browder et al., 1989; Ninci, 2019). Practitioners value the attainment of meaningful student achievement, relative to various instructional goals, more than verification of their instruction as the sole source of changes in behavior (Riley-Tillman et al., 2020). Systematic approaches to visual analysis (e.g., Lane & Gast, 2014) has the potential to reduce disagreement among observers regarding the quantifiable aspects of a line graph but do not aid in determining the magnitude or practical significance of changes in trend.

Procedural descriptions of the split-middle method and similar approaches generally do not include guidelines related to the classification of trend magnitude. Likewise, indicators of treatment effect commonly employed as supplements to visual analysis (e.g., percentage of nonoverlapping data) usually assess the extent of nonoverlap between data in baseline and treatment conditions rather than the magnitude of effect (Wolery et al., 2010). Consequently, such metrics do not represent the best approach to guiding practice or assessing the instructional value of interventions. Tools for deriving suitable, data-based objectives and evaluating the effect of an intervention in the context of progress needed for the attainment of objectives have historically varied with the types of graphs employed in practice.

## Comparisons of Linear and Ratio Graphs

Notwithstanding historical attempts to disseminate idiosyncratic data-based decision making rules for typical linear graphs (e.g., Browder et al., 1989), precision teaching (PT) has produced behavior analytic literature concerning practitioner-oriented data-based decision making (PT; see Binder, 1990; Johnson & Street, 2013; Kubina, 2019). PT emphasizes the importance of changes in performance and alters instruction and other behavioral interventions based on the frequent assessment of progress relative to quantified targets (Kubina, 2019). Yet PT uses the standard celeration chart, a formalized example of a ratio graph. The ratio graph depicts trends without the need for complex calculations. Additionally, practitioners have historically applied PT graphing and decision making procedures to a wide array of behaviors. The increasing demand for data-based decision making (e.g., Fuchs et al., 2021) warrants a closer inspection of how linear and ratio graphs may contribute to visual analysis.

**Figure 1.**
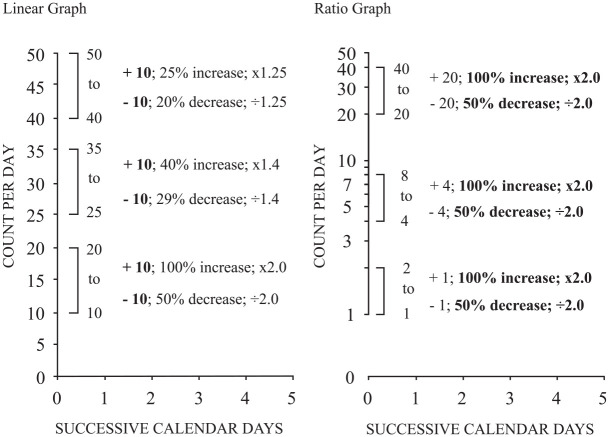
A comparison of a linear and ratio graph. *Note*. Bolded values represent how each graph creates quantitative equivalencies based on visual representation of the distance between two values.

Linear and ratio graphs arguably facilitate different objectives based solely on their construction features, most notably the vertical axis. Linear graphs provide a clear view of *absolute change* and excel at visually presenting commensurate amounts of change to the graph reader. Yet absolute changes do not promote equal ratios of change. Figure 1 displays three comparable magnitudes of change. The space allocation for all three intervals appears the same because all three add or subtract 10 depending on the value and direction of movement (e.g., 25 *+* 10 = 35 or 35–10 = 25). The relative change expressed as a percent growth or decay and a multiplier or divider indicates unequal changes for the three different change distances though visually, they all appear identical. Linear graphs represent how the sheer number of counted units of behavior change have occurred but not the degree of growth or decay.

Conversely, a ratio graph has relative change as its primary feature. Equal distance of space between two values on the vertical axis represents an equal ratio of change and display data changing relative to one another. Figure 1 shows three equal distances between two values on the vertical axis (i.e., 1–2, 4–8, and 20–40). Additively the values of +1, + 4, and + 20, respectively appear visually equivalent because all have the same ratio or proportion of change (Schmid, 1986, 1992). Therefore, the ability to view proportional change relative to an initial rate of response represents one of the primary benefits of ratio graphs. The emphasis on proportional change in visual presentation prevents concealing the significance of nominally small performance changes and overstating the importance of nominally large changes.

A series of data points graphed across time also changes according to the dictates of linearity. A linear graph has its basis in a cartesian coordinate system, and the trend line delineates change based on a slope-intercept equation, y = mx + b. However, the use of an equal-interval scale based on the range of participant responses also represents a primary drawback of linear scaling. The graph allows for easy determination of the direction of trend yet cannot conveniently facilitate the precise quantification of trend (Kinney et al., 2022).

In contrast, ratio graphs feature vertical scales based on a multiply/divide scale and logarithms with a slope determined by log y = mx + log b (Giesecke et al., 2016; Prochaska & Theodore, 2018). The slope on a ratio graph demonstrates growth or decay. The steepness of the slope communicates how fast a quantity changes (Schmid, 1992). A horizontal slope has no growth changing at 0% or ×1.0. Behavior moving upward or downward at a uniform rate will show up as straight and have a percentage change and multiplier/divider associated with the values. The values of growth or decay will depend on the graphed quantities. There exist many differences between linear and ratio graphs which fall beyond the scope of the present discussion; interest readers may wish to examine historical and current sources showcasing the benefits of ratio graphs (Fisher, 1917; Giesecke et al, 2016; Griffin & Bowden, 1963; Harris, 1999; Schmid, 1986, 1992).

As an engineering student, Lindsley created Precision Teaching (PT) and featured the standard celeration chart (SCC) in his system as the driver for data display and decision making. Lindsley based the SCC on Skinner’s use of a standard visual display (i.e., cumulative response recorder) and the benefits of a ratio graph (Lindsley, 1991; Potts et al., 1993). The paper SCC comprises a standard ratio graph that displays data up to 140 days and covers values ranging from 1 per day to 100 per minute or the full range of observable behavior (Kubina & Yurich, 2012). The SCC shows celeration or a measure of growth depicting the change in responding over a period of time (Johnston et al., 2020; Pennypacker et al., 2003).

PT practitioners have established a framework for data-based decision making founded on the SCC and the extent of change over time. From the 1970s to the present, PT researchers and teachers established guidelines that indicated when to change or continue an intervention. One such rule involved using a celeration line, or graphic representation of the change in the rate of behavior over time, to determine if progress meets the change-across-time value (Liberty, 2019; White, 1984). A celeration aim value of ×1.5 means the line represents an increase of 50% per week (Johnston & Street, 2013). Therefore, a decision rule could state, “For a celeration of ×1.5 or greater, continue the intervention. For a celeration less than ×1.5, make a change.” Decision rules based on a quantified value has facilitated objective, clear, data-based actions for chart users (Johnson & Street, 2013; Kubina, 2019).

Despite apparent differences in the properties and application, few studies have empirically evaluated the differences in linear and ratio graphs (Kinney et al., 2022). The bulk of the literature suggests the decision to use a linear or ratio graph should be left to practitioner preference due to minor differences in the effect of graph type on student achievement (Fuchs & Fuchs, 1986) or the interpretation of data features (e.g., trend, level; Knapp, 1983; Bailey, 1984). Kinney et al.’s (2022) comparison of practitioner performance on multiple tasks using linear and ratio graphs supports the notion that different displays may lend themselves to different tasks. Raters (*n* = 74) more accurately identified worsening or improving trends using ratio graphs, whereas linear graphs resulted in higher levels of performance on tasks such as identifying or plotting specific points in a data series. Evidence supporting the use of linear graphs in identifying trend provides some support for using ratio displays in instructional decision making. However, the extent to which either linear or ratio graphs facilitate practitioner decision making, particularly in the context of the forms of analysis typically associated with the two displays, remains unclear.

## Experimental Questions

Much SCED research emphasizes the analysis of linear graphs and places a premium on level. The extent to which the conventional visual analysis of linear graphs facilitates more nuanced decision making based on trends that, though positive, may not indicate the effectiveness of treatment remains unclear. In contrast, ratio graphs and decision making rules may result in more consistent, confident assessments of data patterns and treatment decisions among practitioners due to the addition of quantification and objective guidelines. As they receive explicit training in visual analysis and routinely analyze data in practice, applied behavior analysts may represent an ideal population to evaluate the efficacy of procedural variations in visual analysis. The present study compared the decision making of applied behavior analysts based on the graphical displays and decision-making rules that typically accompany linear and ratio graphs. Specific questions included:

1. To what extent does agreement concerning the magnitude of data trends (e.g., low, high) and appropriate treatment decisions (e.g., change, maintain) vary based on the types of graphs used and their accompanying decision rules?
2. How does graph type influence efficiency of decision making (i.e., speed at which participants respond)?
3. How does graph type influence behavior analysts’ confidence in their evaluations of trend magnitude and treatment decisions?

## Method

### Participants and Settings

We recruited participants from multiple behavior analytic service providers across the northeastern United States. Cooperating administrators within the organization distributed an email with a survey link to professionals. The email indicated that (a) participation would require approximately 30 minutes, and (b) respondents would have the opportunity to participate in a drawing for a $20 gift card. Fifty-one participants agreed to participate and completed the survey in its entirety. Specific details concerning participants’ credentials, practical experience, and data interpretation methods appear in Table 1. An a priori power-analysis conducted in *G-power* indicated that a sample of 42 participants would be sufficient to detect a small effect size (e.g., *r* = .3) at the recommended level of statistical power (.80).

**Table 1.** Participant Demographics.

Characteristics	*n*	%
Gender		
Female	35	66.04
Male	19	33.96
Certification level		
BCaBA	66	81.48
BCBA	10	12.35
BCBA-D	4	4.94
Not certified	1	1.23
Year of experience		
0-5	29	54.72
6-10	13	24.53
11-15	5	9.43
16+	6	11.32
Work setting^a^		
Administration	18	27.27
Clinical	21	31.82
Higher education	2	3.03
Home	9	13.64
Schools	16	24.24
Employment status		
Full-time (40 + hour/week)	50	94.34
Part-time (<40 hours/week)	2	3.77
Unemployed	1	1.89
Self-employed	9	18
Retired	0	0
Preferred method to graph/Interpret data^a^	17	34
Home-made (e.g., excel)	39	39
Visual analysis	39	39
Program-made (e.g., Chartlytics)	15	15
Celeration lines	7	7

*Note. N* = 53.

a Reflects the number and percent of participants selecting “yes” to each option. Survey question allowed for multiple answer per participant creating subtotal greater than 53 (i.e., total number of participants).

### Instrument

#### Graph generation

**Figure 2.**
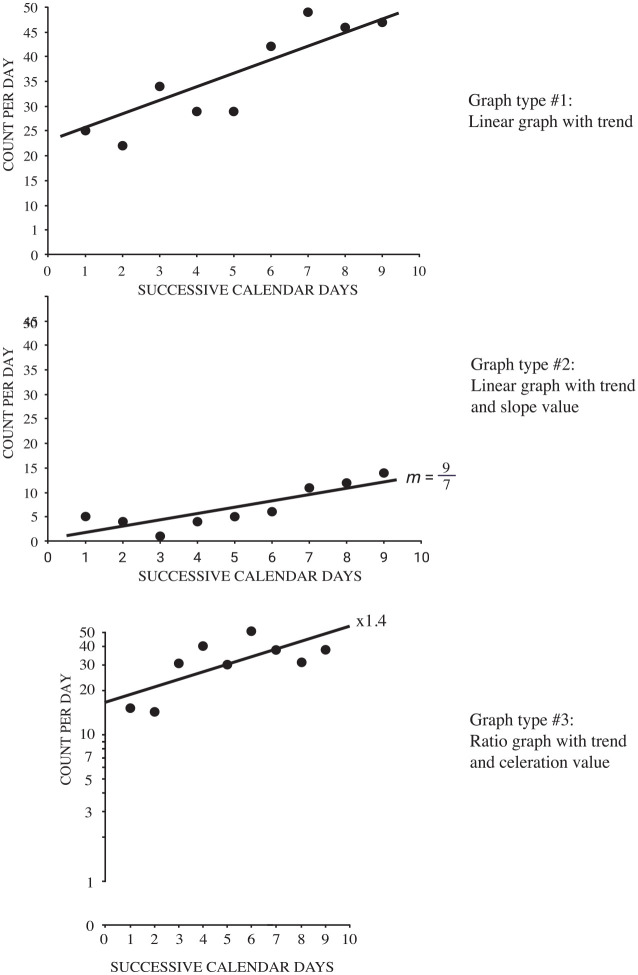
Three different graph types presented to participants.

We used *Adobe Illustrator* and *Microsoft Excel* to generate the graphs. The first author created blank graph templates which showed successive calendar days on the horizontal axis and either an equal-interval scale (*linear graphs*; that is, distance moving up or down on the vertical axis depict additive or subtractive change) or a ratio scale (*ratio graphs*; that is, distance moving up or down on the vertical axis depict multiplicative or divisional change) on the vertical axis (See Figure 2 below for examples of each type graph type). Each graph included nine data points displayed in a positive linear relationship. The pattern of the data points, though, remained appeared identical to one another in each of the three conditions: linear with no slope value, linear graph with a slope value, ratio graph with a celeration value. For the linear graphs with a slope value, the values ranged from 4/7 to 5 2/7 (i.e., slope expressed as rise over run and in the form of fractions and mixed numbers). For the ratio graphs, the celeration values ranged from ×1.1 to ×1.6.

To ensure that the physical characteristics of data paths presented in each condition did not influence responding, we assessed the gradient of the slope, in degrees, of lines in the ratio (*M* = 19.50; Range = 6–35; *SD* = 9.64) and linear/slope conditions (*M* = 19.25; R = 4–36; *SD* = 9.64). Results of an independent sample t-test identified no significant differences in line gradient across conditions (*t*[14] = .04, *p* = .962, *d* = .02). Furthermore, we maintained all data meet a variability standard which included low variability as measured on the ratio graphs (i.e., ×2.5 to ×4 total variability) with a corresponding identical physical distance for variability ranges on linear graphs. And last, we controlled for level by have the overall level for each graph containing and equal distribution of low, medium, and high levels (i.e., range from 5 to 41).

#### Survey

We collected demographic questions, graphs, and related rating scales with a single electronic survey using *Qualtrics*. For demographic items (Table 1), participants completed several items related to their demographic characteristics, including their identified gender, year of experience, certification status, and general work responsibilities. Additional items concerned the approaches respondents used in analyzing graphed data (e.g., visual analysis, split-middle method, celeration lines; Ledford & Gast, 2018). We further assessed respondents’ level of familiarity with approaches to data interpretation using a 4-point Likert-type scale, with “1” indicating no familiarity and “4” indicating high familiarity with a specific method (Table 2).

**Table 2.** Types of Graphs/Methods Participants Use to Interpret Data.

Graphs/Methods	Not at all familiar		Slightly familiar		Moderately familiar		Very familiar	
	*n*	%	*n*	%	*n*	%	*n*	%
Split-middle method	32	60.38	13	24.53	7	13.21	1	1.89
Visual analysis	1	1.89	4	7.55	10	18.87	38	71.70
Home-made	2	3.77	1	1.89	8	15.09	42	79.25
Program-made^a^	12	23.53	15	29.41	12	23.53	12	23.53
Celeration lines^b^	7	13.46	27	51.92	15	28.85	3	5.77

*Note. N* = 53.

a *N* = 51.

b *N* = 52.

After completing the previously described items, participants viewed six instructional videos (each approximately 60 seconds): (1) *Visual Analysis and Decision Rules Instruction* (0:37); (2) *Within and Between Condition Analysis* (0:40); (3) *Estimating and Quantifying Slope* (2:52); (4) *Differences Between Ratio and Linear Graphs* (0:50); (5) *Decision Making for Linear Graphs* (0:46); and (6) *Decision Making for Ratio Graphs* (0:30). The videos explained basic components of visual analysis, how to estimate slope, how to read slopes with either a slope value (linear graphs) or a celeration value (ratio graph), and accompanying decision-making rules (Links to the videos available from the first author). A sample multiple-choice quiz followed each video to assess comprehension and familiarize participants with survey items. Participants repeated incorrect sections until achieving 100% accuracy. We did not include sample items in the analysis.

For each graph, respondents described the trend associated with the data path (e.g., a specific value for ratio graphs “low,” “medium,” or “high” for linear graphs with and without slope values). The difference in the trend identification tasks varied based on the graph type and their associated rules. For ratio values, practitioners only needed to determine the objective celeration value corresponding with change over time, which indicated a specific treatment action. The quantitative value (e.g., ×1.2, ×1.6) reflects a standard rate of change across any trend regardless of level or variability; a characteristic unique to ratio graphs and a means of fostering objectivity). To analyze linear and slope graphs, respondents had to make a qualitative determination regarding the change in performance of assessment sessions (i.e., standard practice in the field and as specified in behavior analytic textbooks).

As with many published SCED, linear graphs did not have any form of quantitative information regarding the characteristic of the line. Slope graphs depicted the slope of the line as a fraction, whole number, or mixed number to control for the possibility of quantitative information improving decision making or improving confidence. Unlike the celeration values for ratio graphs, the ratios included with slope graphs did not correspond with historical rules for interpretation. Respondents then indicated whether they would continue or change a hypothetical treatment based on the performance depicted in the data path. To protect against order effects (i.e., possibility responses varied based on the order of completion), respondents received items in a random order as arranged through *Qualtrics*.

### Dependent Variables

The present study had six dependent variables. Two dependent variables concerned the extent of each respondent’s agreement regarding trend and treatment decision. We calculated agreement for each participant by determining a respondent’s answer for a specific item, determining the number of respondents who selected the same response, and dividing the number by the total number of responses. We then multiplied the number by 100 to yield a percentage agreement score. The remaining dependent variables concerned the respondents’ confidence in their ratings for trend and treatment decisions. Respondents indicated their confidence in each decision using a 6-point Likert-type scale, with “1” representing the lowest level of confidence and “6” representing the highest level of confidence.

Additionally, we determined the efficiency of participants’ responses using two hidden timing questions in the *Qualtrics* survey. The first timer measured initial response time as the number of seconds between the question section appearing on the screen and the participants’ first answer selection (i.e., latency). The second timer measured participants’ total response time as the time between the section loading and the participants’ selection of the “Next” button at the bottom of the screen. We averaged participants’ initial response time and total response measures for each graph type.

### Analysis

We evaluated differences in the within-subject factor of graph type (Ratio, Linear, Slope) for all variables using the Friedman Test, a non-parametric alternative to an ANOVA of repeated measures data. In the event of a significant finding, we examined the significance of comparisons between individual groups using the non-parametric Wilcoxon signed-rank test. Effect sizes were determined using Kendall’s W (for Friedman Test) and Pearson’s *r* (for Wilcoxon signed-rank test), with scores exceeding .5 representing a large effect, scores between .49 and .3 representing a moderate effect, and scores between .29 and .1 representing a small effect. We observed a significance level of *p* = .05 for the Friedman Test. However, we adjusted the significance level for pairwise comparisons involving dependent variables using the Benjamini-Hochberg procedure, with a false-discovery rate of 5%. Survey logic did not permit respondents to skip questions, and we discarded surveys in which respondents discontinued the survey prior to completion; thus, the analysis did not need to account for missing data. All analyses were performed in SPSS.

### Response Rate and Reliability

The survey was disseminated to potential participants described above (i.e., multiple behavior analytic companies). Of those, 81 began the survey. A total of 51 eligible professionals completed the survey in its entirety. We obtained internal consistency data related to confidence scales and decision making using Cronbach’s alpha. Results for scales related to trend assessment confidence (24 items; α = .975) and decision making confidence (24 items; α = .980) were acceptable.

## Results

### Agreement

**Figure 3.**
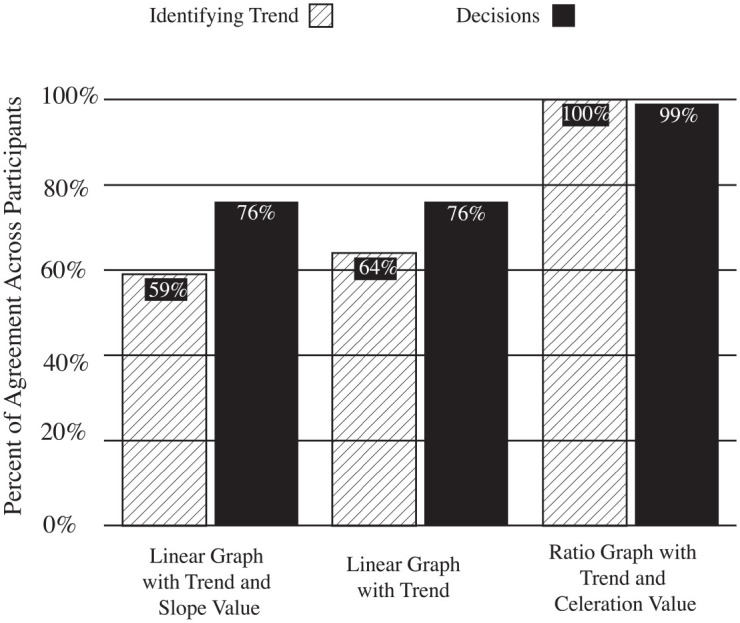
A column graph displaying agreement identifying trend and decision.

Descriptive statistics for agreement measures appear in Table 3. The column graph in Figure 3 graphically portrays participants’ percent agreement identifying trends in each condition and their subsequent treatment decision. Figure 3 shows respondents agreed more frequently on trend on ratio graphs than on either linear or linear plus slope value graphs. Participants agreed the least when viewing data on linear graphs with the slope value assigned to a trend. The participants then made treatments decisions based on their assessment of data and a decision-making rule. The results show both linear graphs with and without slope values evoked the same level of agreement. The ratio graph decision-making rule produced almost the same agreement as participants during the identifying trend condition, 99% versus 100%, respectively.

**Table 3.** Descriptive Statistics for Measures of Confidence, Agreement, and Efficiency across Graph Type.

Graph	Mean confidence (Range/SD)		Mean agreement (Range/SD)		Mean efficiency (Range/SD)	
	Trend	Decision	Trend	Decision	Initial response	Total response
Ratio	5.17 (2.50–6.00/1.10)	5.24 (2.50–6.00/1.05)	100 (-/-)	97.48 (68.38–99.00/5.24)	4.88 (0.63–19.50/3.72)	18.34 (10.55–43.69/7.67)
Linear	4.54 (2.13–6.00/.95)	4.82 (2.25–6.00/1.01)	65.85 (42.50–74.38/7.52)	82.279 (63.25–99.00/6.26)	7.92 (0.73–34.65/6.53)	22.20 (8.31–68.11/11.92)
Slope	4.61 (2.00–6.00/.94)	4.83 (2.00–6.00/1.01)	66.26 (44.88–75.50/9.37)	83.179 (61.00–87.00/6.31)	12.22 (1.02–118.94/30.58)	25.85 (9.89–131.03/21.29)

*Note*. Confidence ratings based on a 6-point Likert-type scale, with 1 representing low confidence. Agreement represents a percentage of responses in which a participants identified the same choice in regard to the assessment of trend or instructional decisions. Response time for efficiency items presented in seconds.

We analyzed data using a Friedman Test with a within-subject factor of graph type (Ratio, Linear, Slope). Results indicated a large, significant effect of graph type on agreement for trend, χ^2^(2) = 78.157, *p* < .000, *r* = .766), and decision making, χ^2^(2) = 71.892, *p* < .000, *r* = .705. In order to determine the difference between pairs, we conducted pairwise comparisons for each type of graph. Results revealed moderate, significant differences in respondent agreement for trend between the ratio and linear graphs at an adjusted significance level of .005 (*Z* = −6.217, *p* <* *.000, *r* = .439). We observed additional moderate, significant differences between agreement for trend in the ratio and slope value graphs at an adjusted significance level of .002 (*Z* = −6.219, *p* <* *.000, *r* = .440). We did not observe significant differences in agreement on trend for the linear and slope value graphs (*Z* = −.619, *p* = .536, *r* = .044)

Pairwise comparisons also revealed similar differences in agreement on decision making. Results indicated moderate, significant differences in respondent agreement on treatment decisions for ratio and linear graphs at an adjusted significance level of .011 (*Z* = −5.787, *p* < .000, *r* = .409). We also observed moderate, significant differences between agreement for treatment decision in the ratio and slope value graphs at an adjusted significance level of .008 (*Z* = −5.908, *p* < .000, *r* *=* .418). Results revealed significant differences in treatment decision agreement between slope and linear graphs at an adjusted significance level of .036 (*Z* = −2.234, *p* = .026, *r* *=* .158).

### Confidence

**Figure 4.**
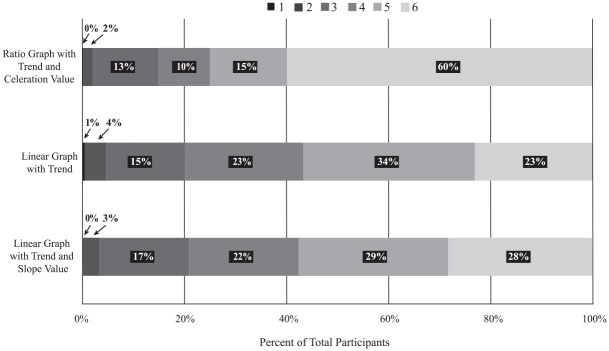
A column graph showing efficiency analyzing data on different graph types.

Descriptive statistics for confidence appear in Table 2, while a 100% stacked bar graph (Figure 4; Harris, 1999) provides a granular view of confidence ratings. In general, respondents reported higher confidence levels for trend and treatment decision ratings on ratio graphs relative to the other graph types. Comparing “completely confident” ratings across the three graph types, the ranges for percent-of-the-whole for ratio graphs plus celeration value, linear graph with trend, and linear graph with trend and slope value came to, respectively, 60%, 23%, and 28%. The total confidence rating averages for ratio graphs appeared 2.6 times higher than linear graphs with trend, and 2.1 times higher than linear graphs with trend and slope value.

Results of the Friedman Test indicated moderate, significant effects of graph type on confidence in ratings for trend χ^2^(2) = 37.780, *p* < .000, *r* = .370, and decision making, χ^2^(2) = 27.445, *p* < .000, *r* = .269. Results of pairwise comparisons related to confidence in trend revealed moderate, significant difference between ratings on ratio and linear graphs (*Z* = −4.885, *p* < .000, *r* = .339) as well as ratio and slope graphs (*Z* = −4.328, *p* < .000, *r* = .312) at significance levels of .013 and .019, respectively. Analyses of decision confidence ratings indicated moderate differences between ratio and linear graphs at an adjusted significance level of .027 (*Z* = −3.994, *p* < .000, *r* = .282); likewise, results suggested a moderate difference in confidence on decisions regarding ratio and slope graphs at an adjusted significance level of .025 (*Z* = −4.051, *p* < .000, *r* = .286). We did not observe significance differences on confidence ratings related to trend or decision making between linear and slope value graphs (*Z* = −.949, *p* = .343, *r* = .067; *Z* = −.232, *p* = .817, *r* = .016).

### Efficiency

**Figure 5.**
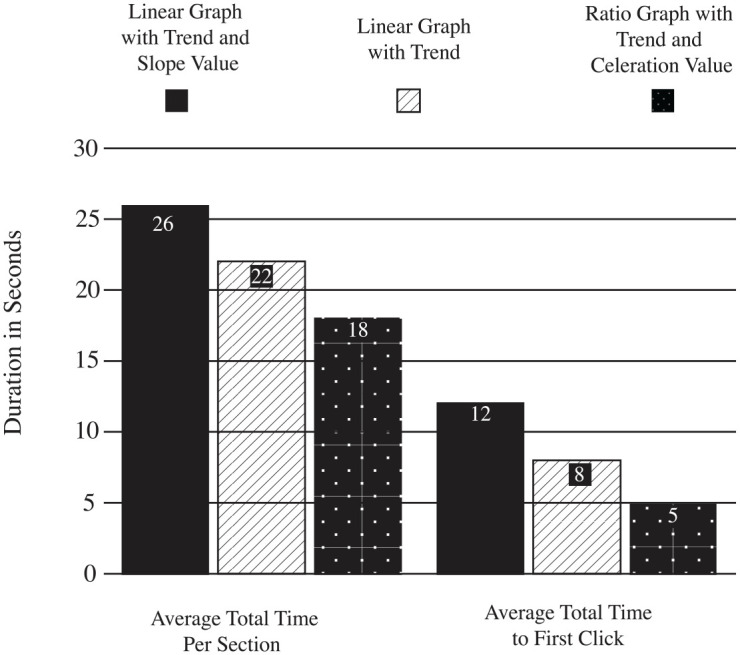
A column graph showing efficiency analyzing data on different graph types.

Descriptive statistics for efficiency appear in Table 2. Figure 5 presents a column graph showing the participants’ average duration in seconds for the total time for each section and the first click after seeing a response item (i.e., a single instance of the three graph types). The results show participants clicked on their first answer most quickly when presented with a ratio graph and a celeration value. The second fastest click occurred with the linear graph with a trend, while the linear graph with a trend and slope value fostered the longest time to click. Total time on each section showed a similar order for efficiency. Participants spent the least amount of time with ratio graphs with a celeration value, more time for linear graphs with a trend, and the most time for linear graphs with a trend and slope value.

Results of the Friedman Test indicated moderate, significant effects of graph type on efficiency for initial responses, χ^2^(2) = 34.980, *p* < .000, *r* = .343, and a small effect on total response time, χ^2^(2) = 12.118, *p* = .002, *r* = .119. Results of pairwise comparison related to initial response revealed significant differences between responses on ratio and linear graphs (*Z* = −4.227, *p* < .000, *r* = .591) as well as ratio and slope graphs (*Z* = −4.771, *p* < .000, *r* = .668), with adjusted significance levels of .022 and .017, respectively. For total response time, pairwise comparisons likewise revealed significant differences between ratio and linear graphs (*Z* = −2.925, *p* = .003, *r* = .409) at an adjusted significance level of .033 as well as ratio and slope graphs (*Z* = −3.946, *p* < .000, *r* = .552) at an adjusted significance level of 031. We did not observe significant differences between initial (*Z* = −1.509, *p* = .131, *r* = .211) or total response times (*Z* = −.591, *p* = .555, *r* = .082) for linear and slope graphs.

## Discussion

The current study examined the effect of different graphic displays and their associated interpretation rules on assessments made by applied behavior analysts. A within condition analysis can focus on many aspects of the data, such as the number of data points, trend, variability/stability, level, outliers, and changes to trend within a condition (Barton et al., 2018; Cooper et al., 2020; Parsonson & Baer, 1978, 1992). We focused mainly on trend while controlling for variability/stability and level. Ratio graphs with an objective celeration value (i.e., Figure 2, graph type 3) resulted in a higher agreement among analysts regarding the magnitude of trend and the decision to change or maintain treatment compared to linear graphs requiring purely subjective appraisals of magnitude (i.e., graph type 1) or accompanied by a slope value (i.e., graph type 2). Results further suggest that professionals trained in visual analysis (i.e., behavior analysts) assessed graphs more efficiently and reported higher degrees of confidence in their assessment of trend and related treatment decisions when provided with ratio graphs.

The literature has repeatedly demonstrated the difficulty of reliably describing presented data patterns using visual analysis (e.g., Danov & Symons, 2008; DeProspero & Cohen, 1979; Ninci et al., 2015; Ottenbacher, 1993; Wolfe et al., 2016). Although some exceptions suggest high reliability can occur under certain conditions (e.g., expert visual analysts; Kahng et al., 2010), the present study found behavior analysts with years of experience and high familiarity with visual analysis achieved low agreement (65.85%) when examining trend data under typical conditions. The data for linear graphs with trend represented how the behavior analytic field currently engages in visual analysis; the graph reader inspects the trend and subjectively determines if the slope fits a low, medium, or high category (Kennedy, 2005). As such, the mean agreement appeared very close to other studies that employed similar methods (e.g., 67%; Normand & Bailey, 2006).

In contrast, the present results show quantifying trend with a celeration value, and providing objective guidelines for interpretation offers a consistent means for determining the magnitude of behavioral change. One hundred percent of the participants identified the celeration value of each trend line correctly. The objectivity of quantifying trend line with a celeration value compared to the subjectivity of qualitatively estimating a trend line explains the difference in results. The results also clarify why previous research did not come to similar conclusions. For example, Bailey (1984) indicated chart type (i.e., ratio, aka semilogarithmic, vs. linear) did not affect ratings of significance for lines of progress. However, Bailey, like many other researchers who compared the two graph types (Lefebre et al., 2008; Kinney et al., 2022; Knapp, 1983; Marston, 1988; Mawhinney & Austin, 1999), asked participants to apply their qualitative judgment of trend lines to both conditions. None of the past research explicitly provided celeration values, one of the main benefits of a ratio graph.

Quantifying the trend with slope created a condition where participants performed most poorly (i.e., 59% agreement). Even though the brief instructional video explained the rise and run and how to interpret trend with slope value, the fractions and mixed numbers appeared to confuse participants when integrating the values with visual analysis. We could not find any published literature in textbooks or behavioral articles with the trend quantified with a slope value. Therefore, the likely combination of no experience and brief instruction did not improve participants' understanding of the trend. Furthermore, using slope values could have confounded participants due to the varied propensity to portray progress, growth, or decay without a constant ratio. A slope value across a series of data 1, 2, 3, 4, 5, 6, 7 and 51, 52, 53, 54, 55, 56, 57 would appear visually similar but represent two progressions seeing 600% and 12% increases across time respectively.

Nonetheless, graphing data on a ratio display does not automatically produce an easily interpretable value. The celeration value found on a standard celeration chart offers a standard, well-defined, easily calculated quantification of change across time when understood. Though not a primary focus of this study, the inability of quantitative values to improve decision making in the absence of standardization represents a potential consideration when considering the merits of systematic approaches to visual analysis based on traditional linear graphs (e.g., Barton et al., 2019; Manolov, 2017; Odom et al., 2018; Shadish, 2014).

We also examined the impact of graph-type on the efficiency of analysis and decision making (Figure 5). Even though most participants (*n* = 65%) reported “not at all familiar” or “slightly familiar” with celeration lines, the overall data suggest the celeration line and value produced the least amount of time determining its direction, value, and appropriate decision contingent upon the decision rule. Efficiency and high agreement suggest two favorable outcomes; (1) behavior analysts will spend less time parsing significant from insignificant trends and (2) past visual analysis problems (e.g., Danov & Symons, 2008; DeProspero & Cohen, 1979; Ninci et al., 2015; Ottenbacher, 1993; Wolfe et al., 2016) have a solution with an objective, quantified trend.

Some researchers may argue we put forward an unfair comparison given that linear graphs, with the exception of the slope sample, lacked quantification and less obvious decision making rules. Yet our study hopes to make such an important point—the existing literature regarding traditional analysis does not provide precise rules regarding the assessment of trend, and the use of linear scales, and variable graphing conventions prevents the formulation of decision making rules based solely on data. Ratio graphs, on the hand, provide a standard quantification of trend such as percentage of growth or rates of change (Devesa et al., 1995; Giesecke et al., 2016). Certain types of ratio graphs like the standard celeration chart have an associated body of literature supporting the circumstances in which data support specific treatment decisions. The use of the latter method might self-evidently be associated with higher practitioner confidence and consensus calls into question the continued use of the former, less precise method. We concede that convention departures must have accompanying evidence supporting new approaches and have conducted the present study in that spirit.

Another fairness argument surrounding the graph type comparison centers on the reduction of ratio data trend into an easily interpretable number. The slope condition suggests the presence of numeric value alone did not increase confidence or agreement among practitioners. Like the intricate methods of trend quantification that accompany visual analysis, and which the sample reported having little use for in practice, the slopes of the line likely served as ineffective tools for decision making because they would ultimately possess the limitations of linear graphs and absolute change. Additionally, a survey asked Behavior Analyst Certification Board verified course sequence instructors how they teach students to estimate trend (Wolfe & McCammon, 2022). Eighty-one percent reported eyeballing or visualizing the data, 51% taught how to fit data with the split-middle line, and 16% of the respondents reported using other methods like linear regression to produce a line of best fit. No data spoke to teaching slope or otherwise quantifying the trend line.

### Limitations

The present study has notable limitations. First, the sample consisted of behavior professionals contacted through their association with clinics in the northeastern US and does not necessarily represent the broader profession. As the study aimed to provide a preliminary, experimental assessment of the impact of graphing conventions (i.e., different graph types and quantified trend), rather than claims regarding the facility of BCBAs more generally, the experiment, therefore, has value despite the use of a relatively small convenience sample. The inclusion of verified “experts” may have resulted in different findings (e.g., Ledford et al., 2019). However, all participants had some form of credential from the national certifying entity for applied behavior analysts. Given the centrality of visual analysis to services delivered by practitioners of ABA, results from certified professionals may have more meaningful implications for the state of practice relative to those of purported experts in the field.

### Future Directions

The control exercised in the current study may expand to cover more varied data characteristics. For example, all variability visually ranged from very stable to stable (or a ×2 to ×4 distance on a standard celeration chart). Increasing the scope of variability and varying level more widely would demonstrate the robustness of experimental findings. Likewise, including more behavior analysts in number, location, and certification level would indicate whether the present study has generality. Extending the study to between condition analyses and different experimental designs could likewise yield interesting findings.

The current findings provide support for the use of ratio graphs and related decision making rules; future scholarship has the potential to verify findings under more authentic conditions. Kuntz et al. (in press) recently found that the presence of lines connecting levels of student performance in baseline to a long-term goal, or airlines, which are frequently used in DBI and more easily constructed in ratio graphs, significantly improved the ability of preservice teachers to make instructional decisions. Similar studies using ratio graphs, which are increasingly acceptable to educators (e.g., Kinney et al., 2022), could provide further evidence of the advantages of alternatives to traditional visual analysis (i.e., subjective appraisal of data). Such studies would preferably compare the utility of displays across entire cases, with much of the responsibility transferred to professionals using standard tools rather than isolated items or tasks mediated by researchers.

Studies asking behavior analysts to estimate trend typically led to low agreement due to several conditions in experience and graph production. First, no behavior analytic textbooks (e.g., Cooper et al., 2020; Mayer et al., 2019) provide standard, precise rules for specifying the degree of a trend’s slope beyond estimation. A representative example states, “Trend can further be characterized by magnitude, and is often described as **steep** or **gradual** and paired with direction (e.g., steep accelerating trend or gradual decelerating trend) bold in original, Barton et al., 2018, p. 185).” Second, almost every published article with a line graph varies from one article to the next in terms of graph construction features such as the length of axes, the proportion of one axis to the other, and scaling of axes (Kubina et al., 2017). The combination of no objective standards or guidance from authoritative sources such as textbooks, journals, or professional organizations and the accepted practice of idiosyncratically constructed graphs promotes a lack of consistent pattern recognition and lower rates of trend identification. Whether through the adoption of standard ratio displays, systematic visual analysis, or more dedicated training, improving the integrity of visual analysis represents a priority for behavior analysis and other disciplines that rely on graphic displays.

## Supplemental Material

sj-pdf-1-bmo-10.1177_01454455221130002 – Supplemental material for Slope Identification and Decision Making: A Comparison of Linear and Ratio Graphs Click here for additional data file. Supplemental material, sj-pdf-1-bmo-10.1177_01454455221130002 for Slope Identification and Decision Making: A Comparison of Linear and Ratio Graphs by Richard M. Kubina, Seth A. King, Madeline Halkowski, Shawn Quigley and Tracy Kettering in Behavior Modification
