# Supplementary material for: Slope Identification and Decision Making: A Comparison of Linear and Ratio Graphs
Source: Behav Modif. 2022 Nov 13;47(3):615–43. doi: 10.1177/01454455221130002 (PMC10150256; doi:10.1177/01454455221130002)
Supplement: sj-pdf-1-bmo-10.1177_01454455221130002 – Supplemental material for Slope Identification and Decision Making: A Comparison of Linear and Ratio Graphs [file sj-pdf-1-bmo-10.1177_01454455221130002.pdf]

Reviewer Concerns	Authors’ Response

Figure 1 doesn't do much for me. It compares interval-level presentation to ratio-scale presentations but it didn't do much to clarify how one presentation displays relative change better than the other. Please think about re-drafting this. Perhaps some reference in the text to S. S. Stevens might help.	We significantly changed Figure 1 based on feedback. We also expanded the text to better communicate the difference between the two graphs. Our intention is not to offer all the reasons for why a ratio graph is better than a linear graph as that would be a paper in its own right. But reviewer 1 makes a a point about indicating some reasons for why a ratio graph could be better suited to analysis and we changed the figure and our text in that light. The new text follows:<br><br>Conversely, a ratio graph has relative change as its primary feature. Equal distance of space between two values on the vertical axis represents an equal ratio of change and display data changing relative to one another. Figure 1 shows three equal distances between two values on the vertical axis (i.e., 1 to 2, 4 to 8, and 20 to 40). Additively the values of +1, + 4, and + 20 respectively appear visually equivalent because all have the same ratio or proportion of change (Schmid, 1986, 1992). Therefore, the ability to view proportional change relative to an initial rate of response represents one of the primary benefits of ratio graphs. The emphasis on proportional change in visual presentation prevents concealing the significance of nominally small performance changes and overstating the importance of nominally large changes.<br><br>A series of data points graphed across time also changes according to the dictates of linearity. A linear graph has its basis in a cartesian coordinate system, and the trend line delineates change based on a slope-intercept equation, y = mx + b. However, the use of an equal-interval scale based on the range of participant responses also represents a primary drawback of linear scaling. The graph allows for easy determination of the

Explain the term,"celeration" better. Maybe discuss the Standard Celeration Chart.	We added a wholes paragraph and blended it into another paragraph to better explain the chart and Celeration.:<br><br>As an engineering student, Lindsley created Precision Teaching (PT) and featured the standard celeration chart (SCC) in his system as the driver for data display and decision making. Lindsley based the SCC on Skinner's use of a standard visual display (i.e., cumulative response recorder) and the benefits of a ratio graph (Lindsley, 1991; Potts et al., 1993). The paper SCC comprises a standard ratio graph that displays data up to 140 days and covers values ranging from 1 per day to 100 per minute or the full range of observable behavior (Kubina & Yurich, 2012). The SCC shows celeration or a measure of growth depicting the change in responding over a period of time (Johnston et al., 2020; Pennypacker et al., 2003).<br><br>PT practitioners have established a framework for data-based decision making founded on the SCC and the extent of change over time. From the 1970s to the present, PT researchers and teachers established guidelines that indicated when to change or continue an intervention. One such rule involved using a celeration line, or graphic representation of the change in the rate of behavior over time, to determine if progress meets the change-across-time value (Liberty, 2019; White, 1984). A celeration aim value of x1.5 means the line represents an increase of 50% per week (Johnston & Street, 2013). Therefore, a decision rule could state, "For a celeration of x1.5 or greater, continue the intervention. For a celeration less than x1.5, make a change." Decision rules based on a quantified value has facilitated objective, clear, data-based actions for chart users (Johnson & Street,

Figures 3 and 5 might look better if the authors used patterns rather than shading.	We added patterns to figure 3 and 5 as per the request of the reviewer.
Figure 4 is far too busy for me. Can you think of better way to display your data?	We completely reformatted figure 4 by simplifying it. It is less busy and we hope much easier view and understand.
The addition of slope and celeration values might be missed by most readers (viewers?) At least, can be larger?	We deleted the values as we recast the figure.
